# Correction: Exploring magnetoelectric nanoparticles for advanced nano-electroporation and drug delivery in interventional cardiology

**DOI:** 10.1039/d6na90057g

**Published:** 2026-07-21

**Authors:** A. Tommasini, G. Suarato, S. Fiocchi, E. Chiaramello, A. Marrella, M. Lenzuni, M. Parazzini, B. Cortese, P. Ravazzani

**Affiliations:** a Dipartimento di Elettronica, Informazione e Bioingegneria, Politecnico di Milano Piazza Leonardo da Vinci 32 Milano 20133 Italy; b Cnr-Istituto di Elettronica e di Ingegneria Dell’Informazione e Delle Telecomunicazioni Piazza Leonardo da Vinci 32 Milano 20133 Italy giulia.suarato@cnr.it; c Interventional Coronary Center, University Hospitals Harrington Heart & Vascular Institute Cleveland Ohio USA; d Fondazione Ricerca e Innovazione Cardiovascolare Via E. Ponti, 49 Milan 20136 Italy; e DCB Academy Via E. Ponti, 49 Milan 20136 Italy

## Abstract

Correction for ‘Exploring magnetoelectric nanoparticles for advanced nano-electroporation and drug delivery in interventional cardiology’ by A. Tommasini *et al.*, *Nanoscale Adv.*, 2025, **7**, 5978–5992, https://doi.org/10.1039/d5na00438a.

The authors regret that Table 1 and Table 2 did not display the intended information in the original article. The corrected Tables 1 and 2 are displayed below.

**Table 1:** Barium titanate shell properties

Property	Symbol	Value	Reference
Density (kg m^−3^)	*ρ*	5700	COMSOL library
Relative permeability	*µ*_r_	1	COMSOL library
Electrical conductivity (S m^−1^)	*σ*	178.5	COMSOL library
Relative permittivity	{*ε*_r11_; *ε*_r22_; *ε*_r33_}	{1115.1, 1115.1, 1251.3}	COMSOL library

**Table 2:** Cobalt ferrite core properties

Property	Symbol	Value	References
Density (kg m^−3^)	*ρ*	5.2 × 10^3^	45, COMSOL library
Relative permittivity	*ε*_r_	10	COMSOL library
Electrical conductivity (S m^−1^)	*σ*	5.2 × 10^6^	COMSOL library
Poisson's ratio	*ν*	0.48	46, COMSOL library
Young's modulus (Pa)	*E*	230 × 10^9^	46 and 47
Saturation magnetization (A m^−1^)	*M*_S_	3.69 × 10^5^	48
Initial magnetic susceptibility	*χ*_0_	3	49, COMSOL library
Saturation magnetostriction (ppm)	*λ*_s_	−200	42 and 46

The conclusions of the article remain unaffected.

The Royal Society of Chemistry apologises for these errors and any consequent inconvenience to authors and readers.

**References**

42 S. Betal, B. Shrestha, M. Dutta, L. F. Cotica, E. Khachatryan, K. Nash, L. Tang, A. S. Bhalla and R. Guo, *Sci. Rep.*, 2016, **6**, 32019.

45 M. Kurian, S. Thankachan, D. S. Nair, A. E. K, A. Babu, A. Thomas and B. Krishna K. T., *J. Adv. Ceram.*, 2015, **4**, 199–205.

46 X. Zhao, M. Feng, M. Liu, J. Hua, J. Ma, L. Wu, H. Xu, A. P. Wang and H. B. Li, *Mater. Res. Lett.*, 2018, **6**, 592–597.

47 S. Kumar, S. K. Meena and R. Jain, *Int. J. Adv. Res. Eng. Technol.*, 2021, **12**, 32–37.

48 C. N. Chinnasamy, M. Senoue, B. Jeyadevan, O. Perales-Perez, K. Shinoda and K. Tohji, *J. Colloid Interface Sci.*, 2003, **263**, 80–83.

49 S. Betal, M. Dutta, B. Shrestha, L. Cotica, L. Tang, A. Bhalla and R. Guo, *Integr. Ferroelectr.*, 2016, **174**, 186–194.

